# The Management and Challenges of Laparoscopic Cholecystectomy in Situs Inversus Abdominalis

**DOI:** 10.7759/cureus.50012

**Published:** 2023-12-05

**Authors:** Francisco Girão de Caires, António Girão de Caires, Priscila Rietsch Flores, Duarte Gil Alves, Rita Camarneiro

**Affiliations:** 1 General Surgery, Centro Hospitalar do Oeste, Caldas da Rainha, PRT; 2 General Surgery, Hospital Central do Funchal, Funchal, PRT; 3 Intensive Care Unit, Centro Hospitalar Médio Tejo, Abrantes, PRT; 4 General Surgery, Hospital Dr. Nélio Mendonça, Funchal, PRT

**Keywords:** cholecystectomy, laparoscopic cholecystectomy, situs inversus abdominals, minimally invasive laparoscopy, hepato pancreato biliary surgery

## Abstract

Left-sided gallbladders are rare anatomical variations and a result of an abnormal embryological process. The most frequent cause for a sinistroposition gallbladder is the presence of *situs inversus*.

We present a case of a 51-year-old male referred to the General Surgery consult due to cholelithiasis with a history of occasional post-prandial abdominal pain in the left hypochondrium and nausea associated with the ingestion of lipid-rich meals. The ultrasound revealed a gallbladder filled with calculous but without inflammatory signs or bile duct dilation, in the sinistroposition. Magnetic resonance imaging (MRI) confirmed and excluded further anatomic variations. The patient underwent a laparoscopic cholecystectomy due to symptomatic cholelithiasis without any complications and was discharged the following day. When faced with a patient with gallbladder/biliary duct disorders associated with *situs inversus,* one must have a high clinical index of suspicion to properly diagnose and the mental agility to adapt and further operate in a mirrored-positioned abdomen.

In these situations the patient should always undergo a prior MRI to determine the correct anatomy of the biliary system and the surgeon should perform an intraoperative cholangiography if any other variations are suspected.

The presence of *situs inversus* thus imposes a surgical and diagnostic challenge. Although rare the surgeon must be aware of this possibility.

## Introduction

Left-sided gallbladders are rare anatomical variations and a result of an abnormal embryological process [1]. The most frequent cause for a sinistroposition gallbladder is the presence of s*itus inversus* [2].

*Situs inversus* is a unique condition in which the orientation of asymmetric organs is a mirror image of normal anatomy. It may be partial, if only one of the abdominal/thoracic cavities is involved, or *totalis* with the lateral transposition of the organs on both compartments [3].

The association of this condition with other disorders make for a challenging diagnosis and management of abdominal pathology [4].

## Case presentation

We present a case of a 51-year-old male referred to the General Surgery consultation due to cholelithiasis with a history of nausea and occasional post-prandial abdominal pain in the left hypochondrium associated with the ingestion of lipid-rich meals.

There was no past medical or surgical history and no history of smoking or alcohol abuse. Physical examination revealed a painless abdomen with dull percussion of the left hypochondrium. No other significant aspects were found.

**Figure 1:**
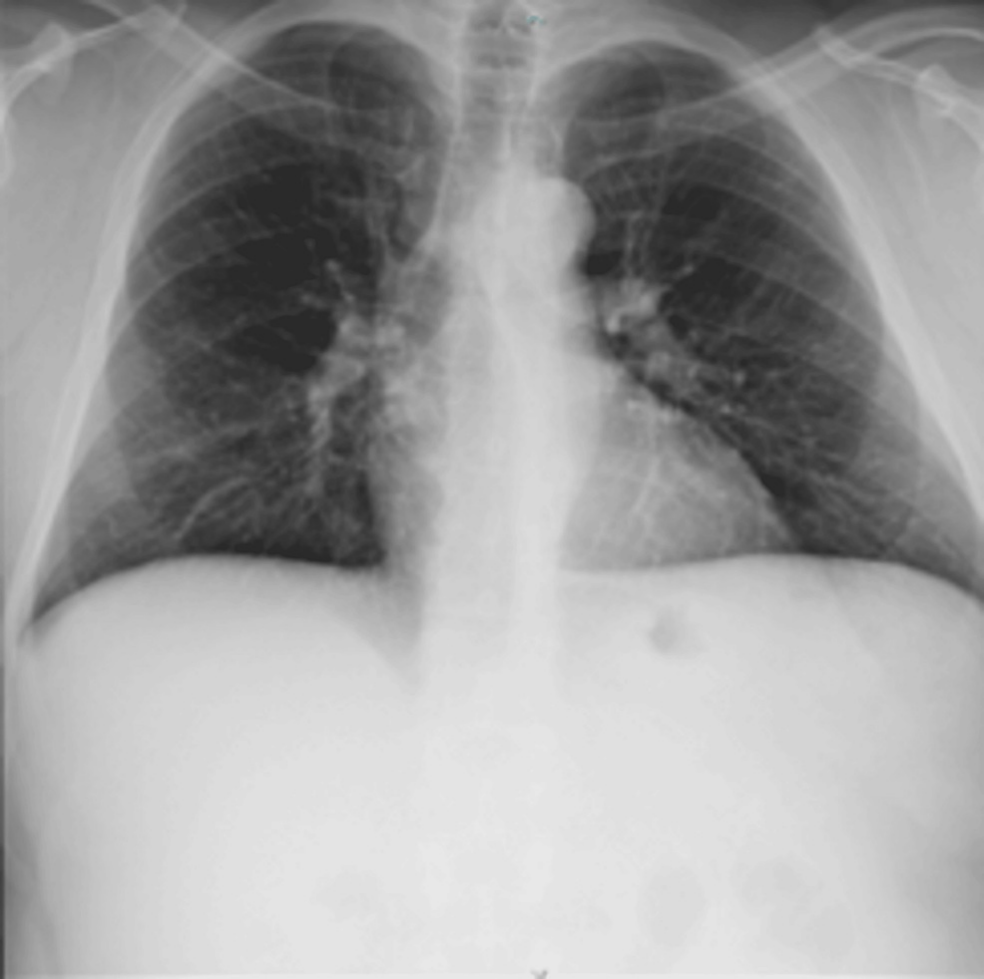
Thoracic X-ray Thoracic X-ray showing normal cardiac position.

Laboratory results were within normal limits and pre-op X-ray showed no abnormalities (Figure 1).

The ultrasound revealed a gallbladder, in the sinistroposition, filled with calculous but without inflammatory signs or bile duct dilation.

**Figure 2:**
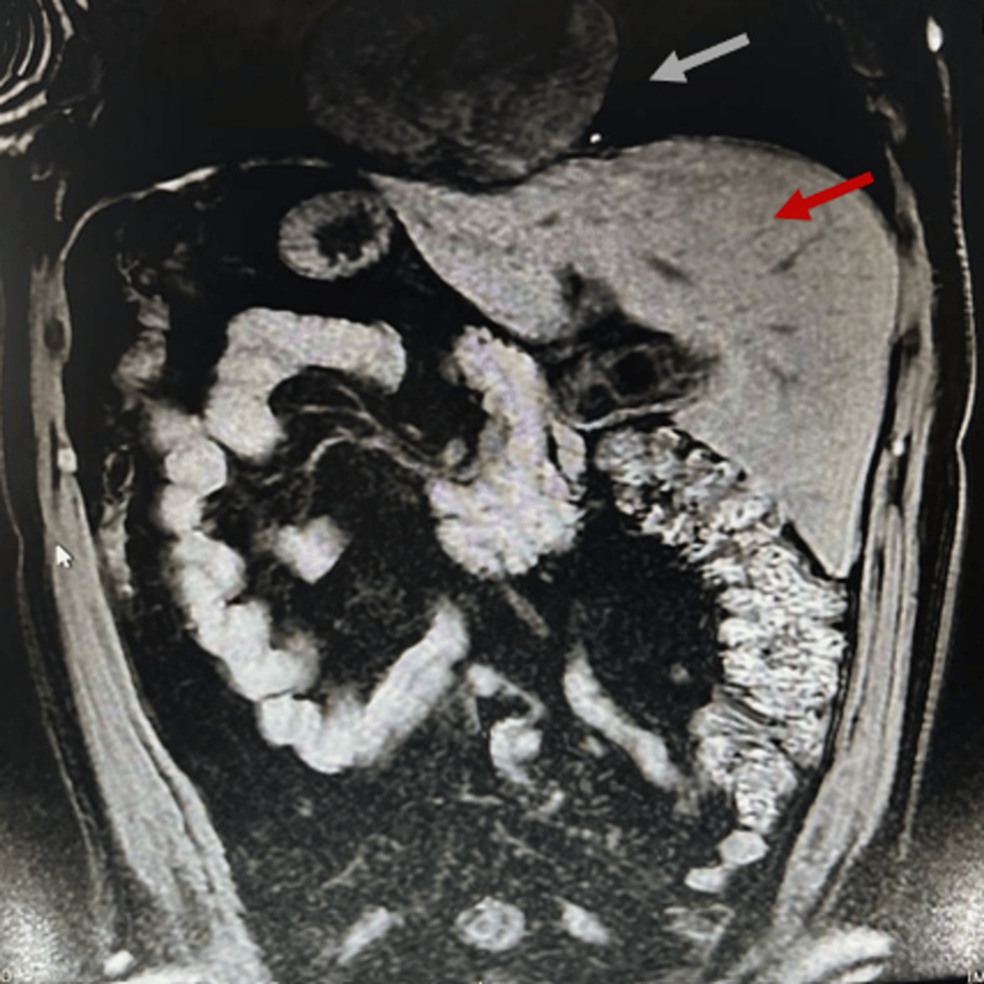
MRI, T1 weighted, coronal plane Grey arrow: Normal cardiac position Red arrow: Liver in the sinistroposition confirming *situs inversus* *abdominalis*.

**Figure 3:**
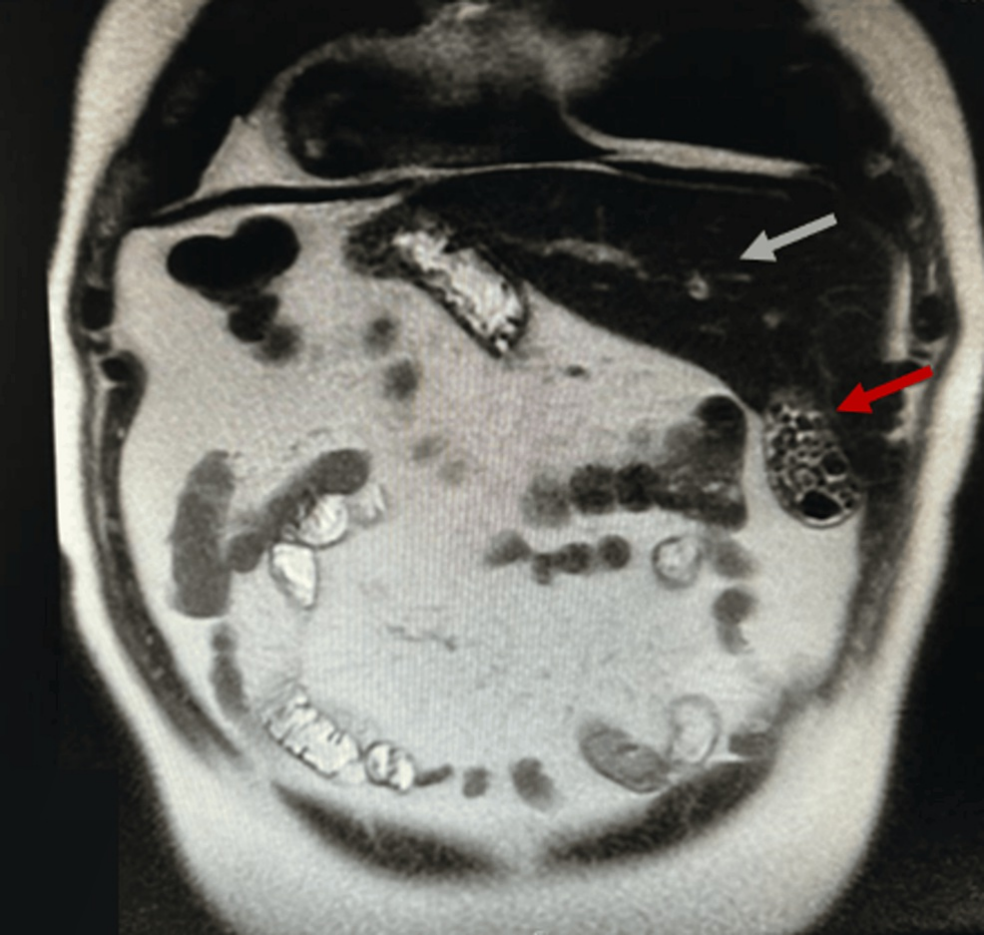
MRI, T2 weighted, coronal plane Grey arrow: Liver in the sinistroposition Red arrow: Subhepatic gallbladder, on the left side of the abdomen

**Figure 4:**
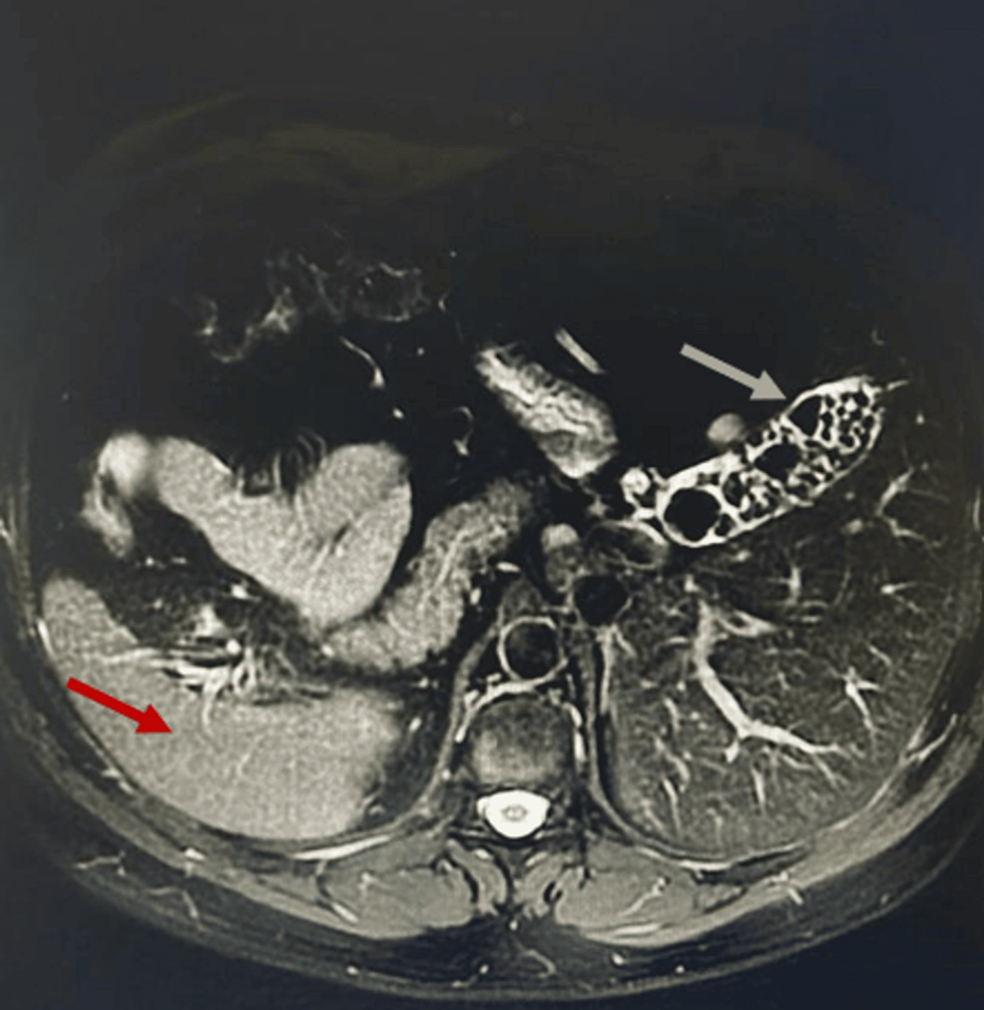
MRI, T2 weighted, axial plane Grey arrow: Calculous gallbladder Red arrow: Right-sided spleen

Magnetic resonance imaging (MRI) confirmed the usual subhepatic position of the gallbladder, but on the left hypochondrium, and ruled out other anatomic variations of the biliary ducts and vascular anatomy (Figures 2, 3, 4).

**Figure 5:**
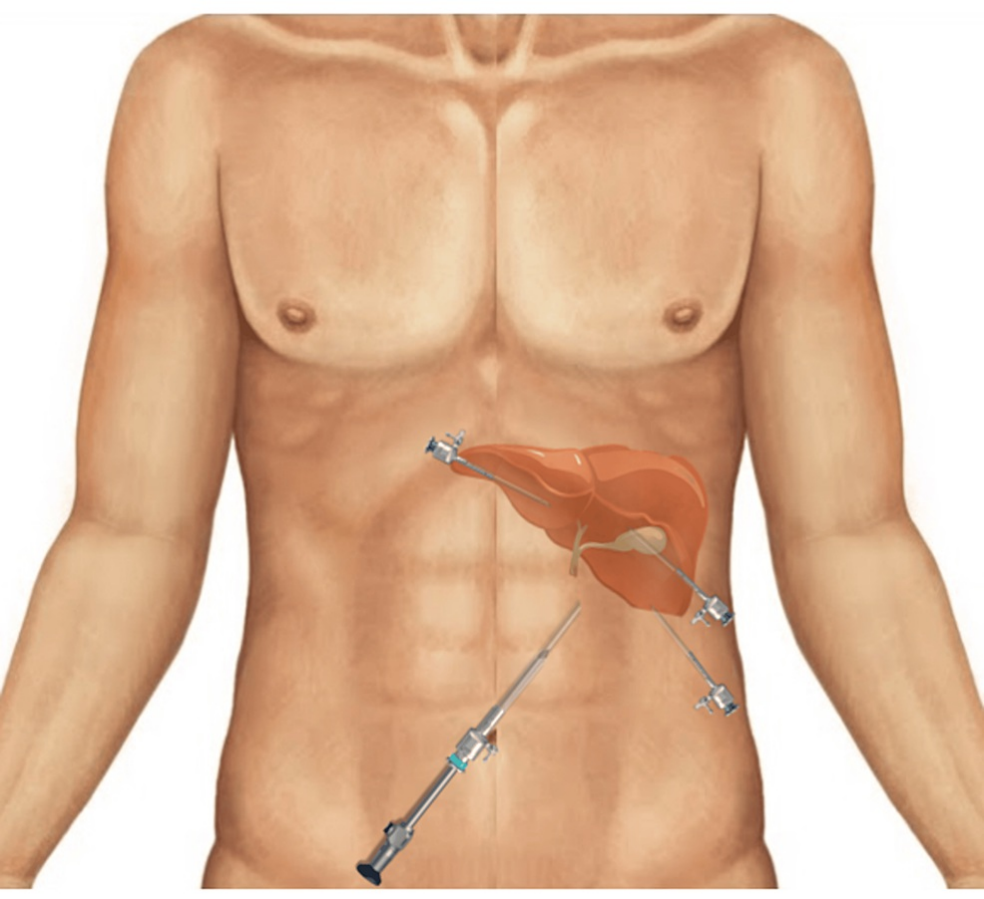
Trocar placement Inverted American technique trocar placement Image credit: Authors

The patient underwent a laparoscopic cholecystectomy due to symptomatic cholelithiasis. With the patient in the supine position, the insufflation of the abdomen was achieved to 12 mmHg with the Veress needle after the umbilical incision. The American technique was used (trocar placement: 10 mm - umbilical; 10 mm - subxiphoid, medial subcostal and lateral subcostal, both on the left side) and a 5 mm optic was inserted in the umbilical port (Figure 5).

**Figure 6:**
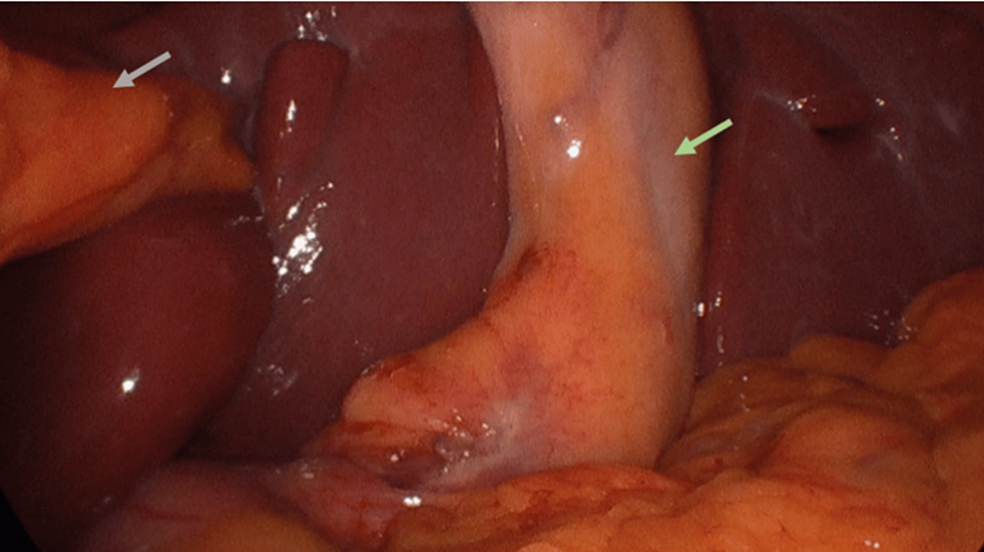
The mirrored image of the gallbladder Grey arrow: Falciform ligament Green arrow: Gallbladder

Laparoscopic survey was performed, confirming *situs inversus* *abdominalis *(Figure 6).

**Figure 7:**
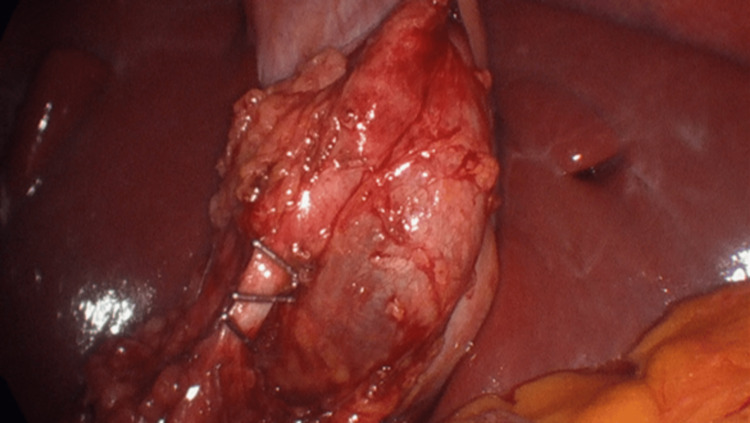
Dissection of Calot's triangle

We proceeded with the dissection of the hepatocystic triangle allowing the identification of the cystic duct and artery (Figure 7).

Mirrored image anatomy of the gallbladder, common bile duct, and cystic duct was observed and, after achieving the critical view of the safety of Strasberg, the two structures were clipped and divided in a standard fashion.

**Figure 8:**
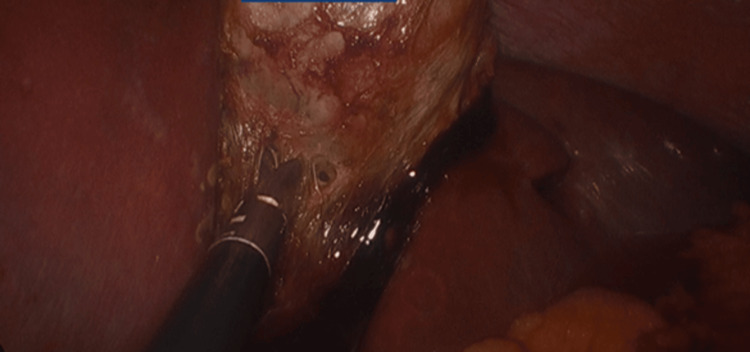
Dissection of the cystic plate

The gallbladder was excised from its plate and removed in an endobag through the umbilical port (Figure 8).

There were no intraoperative complications. The patient was discharged on the following day with an uneventful hospital stay. The pathology report confirmed the presence of chronic cholecystitis which was compatible with our diagnostic hypothesis. Thirty days after the surgery, the main complaints were resolved and the patient was discharged from the General Surgery consultation, confirming the diagnosis.

## Discussion

*Situs inversus* has an incidence of 1:5,000 to 1:10,000 with a slight male predominance and is the most frequent cause of sinistroposition gallbladders [5]. Left-sided gallbladders are not often identified pre-operatively and, because they are associated with biliary and vascular anomalies, the surgeon must be familiar with the variations that he might encounter [1]. In a multicenter study, the prevalence of sinistroposition gallbladders was 0.3% in laparoscopic cholecystectomies [6]. The prevalence of an anomalous bifurcation of the cystic duct from the left hepatic duct in this condition is 5.6% and 14.3% [5]. With the high prevalence of gallstone disease, it is remarkable that less than 40 cholecystectomies in patients with *situs inversus* were reported in the literature during the pre-laparoscopic era [7].

Modern-day MRI has made imaging of the biliary tract faster, with excellent anatomic reproduction of the biliary ducts. MRI/MR-cholangiopancreatography (MRCP) has now become the first-line imaging method for the investigation of this duct system [8]. Intra-operative anatomy that does not appear normal should suggest the possibility of biliary malformations and dissection should proceed with extreme caution [9]. This fact highlights the necessity to achieve a critical view of safety before cutting or dividing tubular structures [5,6].

Whenever there is a poorly defined anatomy, intraoperative cholangiography ought to be performed to detect associated anomalies of the biliary tree [9]. Selective use of intraoperative cholangiography and meticulous dissection can aid in a safe resection [10]. The choice of Palmer’s point to achieve pneumoperitoneum might not be the best option when faced with a mirrored image of the abdominal organs. The success of a minimally invasive cholecystectomy may not be achievable since there is a wide spectrum of possible anomalies associated with left-sided gallbladders [11,12]. As a last resource, the conversion to laparotomy should be taken into consideration before any avoidable complications occur [5].

## Conclusions

When faced with a patient with gallbladder/biliary duct disorders associated with *situs inversus*, one must have a high clinical index of suspicion to properly diagnose and the mental agility to adapt and further operate in a mirrored-positioned abdomen.

In this situation, the patient should always undergo a prior MRI to determine the correct anatomy of the biliary system.

The presence of *situs inversus* thus imposes a surgical and diagnostic challenge and, although rare, the surgeon must be aware of this possibility.

## References

[1] Iskandar ME, Radzio A, Krikhely M, Leitman IM (2013). Laparoscopic cholecystectomy for a left-sided gallbladder. World J Gastroenterol.

[2] Butt MQ, Chatha SS, Ghumman AQ, Rasheed A, Farooq M, Ahmed J (2015). Laparoscopic cholecystectomy for left sided gallbladder in situs inversus totalis. J Coll Physicians Surg Pak.

[3] Akbulut S, Ulku A, Senol A, Tas M, Yagmur Y (2010). Left-sided appendicitis: review of 95 published cases and a case report. World J Gastroenterol.

[4] Benhammane H, Kharmoum S, Terraz S (2012). Common bile duct adenocarcinoma in a patient with situs inversus totalis: report of a rare case. BMC Res Notes.

[5] Janchar T, Milzman D, Clement M (2000). Situs inversus: emergency evaluations of atypical presentations. Am J Emerg Med.

[6] Carbajo MA, Martín del Omo JC, Blanco JI (1999). Congenital malformations of the gallbladder and cystic duct diagnosed by laparoscopy: high surgical risk. JSLS.

[7] Oms LM, Badia JM (2003). Laparoscopic cholecystectomy in situs inversus totalis: the importance of being left-handed. Surg Endosc.

[8] Krausé D, Cercueil JP, Dranssart M, Cognet F, Piard F, Hillon P (2002). MRI for evaluating congenital bile duct abnormalities. J Comput Assist Tomogr.

[9] Gui D, Magalini S, Prete F, Sermoneta D (2002). What's right when the gallbladder's left? A case report. Surg Endosc.

[10] Idu M, Jakimowicz J, Iuppa A, Cuschieri A (1996). Hepatobiliary anatomy in patients with transposition of the gallbladder: implications for safe laparoscopic cholecystectomy. Br J Surg.

[11] Sadhu S, Jahangir TA, Roy MK (2012). Left-sided gallbladder discovered during laparoscopic cholecystectomy in a patient with dextrocardia. Indian J Surg.

[12] Abongwa HK, De Simone B, Alberici L (2017). Implications of left-sided gallbladder in the emergency setting: retrospective review and top tips for safe laparoscopic cholecystectomy. Surg Laparosc Endosc Percutan Tech.

